# Multivariate meta-analysis for non-linear and other multi-parameter associations

**DOI:** 10.1002/sim.5471

**Published:** 2012-07-16

**Authors:** A Gasparrini, B Armstrong, M G Kenward

**Affiliations:** a Department of Medical Statistics, London School of Hygiene and Tropical MedicineLondon, U.K.; b Department of Social and Environmental Health Research, London School of Hygiene and Tropical MedicineLondon, U.K.

**Keywords:** meta-analysis, multivariate analysis, multivariate meta-analysis, non-linear, splines

## Abstract

In this paper, we formalize the application of multivariate meta-analysis and meta-regression to synthesize estimates of multi-parameter associations obtained from different studies. This modelling approach extends the standard two-stage analysis used to combine results across different sub-groups or populations. The most straightforward application is for the meta-analysis of non-linear relationships, described for example by regression coefficients of splines or other functions, but the methodology easily generalizes to any setting where complex associations are described by multiple correlated parameters. The modelling framework of multivariate meta-analysis is implemented in the package mvmeta within the statistical environment R. As an illustrative example, we propose a two-stage analysis for investigating the non-linear exposure–response relationship between temperature and non-accidental mortality using time-series data from multiple cities. Multivariate meta-analysis represents a useful analytical tool for studying complex associations through a two-stage procedure. Copyright © 2012 John Wiley & Sons, Ltd.

## 1. Introduction

Meta-analysis is a standard, well-grounded statistical procedure for combining the evidence from independent studies that address the same research hypothesis [1]. This methodology was developed originally for pooling the results from published observational or experimental studies, for which individual data were not available. Recently, meta-analysis has been described more broadly as a research synthesis method, with the aim of estimating an average association across studies and to explore the degree and sources of heterogeneity [2]. The analytical approach adopted in this context may be described as a two-stage hierarchical procedure: in the first stage, study-specific estimates of the association of interest are derived from individual data, controlling for individual-level covariates; in the second stage, these estimates are combined across studies, optionally exploring the association with study-level predictors. The two-stage approach, a specific form of individual patient data (IPD) meta-analysis, has been shown to be a flexible and computationally efficient method [3] and has been adopted in different contexts: to pool estimates from multiple randomized controlled trials [4]; to combine results from survival models on time-to-event data in multi-centre cohorts [5]; and to synthesize associations from Poisson time-series models in multi-city analyses [6].

The common approach to two-stage meta-analysis consists of summarizing the association in a single parameter estimate from the first stage, optionally controlling for individual-level confounders. This procedure allows standard meta-analytic techniques to be applied. However, complex associations, such as non-linear exposure–responses, are usually described with functions defined by multiple parameters and require more sophisticated meta-analytical approaches capable of handling the multivariate nature of the summary estimates. Multivariate meta-analysis, a method originally developed to pool multiple correlated outcomes in randomized controlled trials [7–9], provides a platform to extend the standard two-stage meta-analytical approach.

The aim of this article is to formalize the application of multivariate meta-analytic techniques to the synthesis of multi-parameter associations from two-stage hierarchical analyses, describing the statistical framework, methodological issues, limitations and research directions. This contribution originates from a commentary published in this journal [10] to the paper by Jackson and collaborators on multivariate meta-analysis [11]. The article also offers the opportunity to describe the implementation in the package mvmeta within the R software [12], designed to perform multivariate meta-analysis and meta-regression in this and other contexts. The document is structured as follows. In Section 2, we introduce an example to illustrate the application of the methodology, consisting of a two-stage meta-analysis of non-linear temperature–mortality associations in 20 US cities. We describe the statistical methodology in the next two sections: in Section 3, we introduce in general terms the first-stage analysis, and we illustrate the modelling framework of multivariate meta-analysis in Section 4, with a specific focus on the setting of multi-parameter associations. We describe the results in Section 5 and emphasize specific methodological issues in Section 6. Finally, we provide a general discussion in Section 7, also reviewing previous research on the topic. The Supplementary Web Appendix contains additional information on the software and the complete R code to replicate the results of the analysis illustrated in Section 5.

## 2. Motivating example

In this section, we describe an example of the application of multivariate meta-analysis for multi-parameter associations. Specifically, we apply the framework to the combination of estimates of the non-linear exposure–response relationship between outdoor temperature and non-accidental mortality, using time-series data from 20 cities in the USA. The example is illustrative, providing a context for the statistical problem, and is not meant to provide substantive evidence on the topic. Subject-specific methodological issues are discussed elsewhere [13]. The illustration is presented in general terms, emphasizing the applicability of the statistical framework beyond the specific research field, first-stage models or multi-parameter functions discussed in this example.

### 2.1. Temperature–health associations

The health effects of exposure to extreme temperatures have been frequently investigated, especially regarding the association with high temperatures and heat waves [14]. Analyses are typically based on a time-series design, comparing averaged daily temperature with aggregated daily outcomes, often mortality or morbidity counts occurring in a specific city. Most studies have reported an increased risk for both cold and hot temperatures and have used a variety of functions to describe such non-linear association in regression models [15]. The association has shown a strong geographical pattern, linked with climatological, socio-economic and demographic factors of each city [16]. This issue has prompted the use of multi-city analyses, where the results from different cities are pooled together, and explanation for differences is sought through reference to study-specific characteristics such as those listed previously. Individual data, corresponding to series of daily observations from different cities, are usually available, and the analytical setting corresponds to the two-stage framework detailed in Section 1. Here the terms *study* and *city* may be used interchangeably to refer to second-stage units.

Different analytical approaches have been proposed for modelling the health effects of temperature in multi-study settings [13]. Traditional methods rely on the simplification of the dependency, assuming a linear association beyond a threshold [17]. An alternative uses non-linear representations such as splines or polynomials but reduces the estimand to a simple comparison between two specific temperatures [18]. Both these approaches are motivated by the need to summarize the study-specific associations in a single parameter for meta-analysis. Such simplifications are, however, based on strong assumptions about the exposure–response shape or, alternatively, only provide a partial picture of possibly complex dependencies. This supports the development of methods allowing the meta-analysis of truly non-linear multi-parameter relationships.

### 2.2. Data

**Figure 1:**
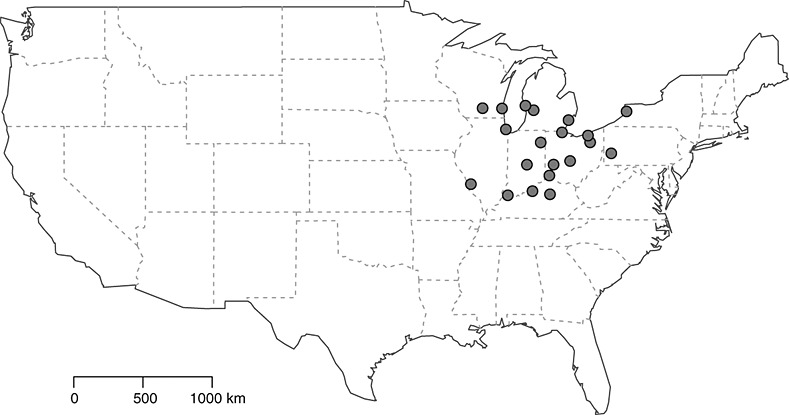
Map of the 20 cities in the USA corresponding to the studies included in the analysis. The cities are located in the industrial Midwest region, as defined in the original National Morbidity, Mortality and Air Pollution Study.

The multi-city time-series data used in this analysis were collected as part of the National Morbidity, Mortality and Air Pollution Study (NMMAPS) (http://www.ihapss.jhsph.edu). This publicly available database contains, among other information, daily series of mortality counts and weather and pollution measurements totalling 5114 observations for the period 1987–2000 in each of 108 cities in the USA. For the purpose of this illustrative example, we restrict the analysis to the 20 cities illustrated in Figure 1, situated in the industrial Midwest region, as defined in the original study.

In addition, the database includes city-level measures of several variables on geographical, climatological, demographic and socio-economic characteristics. A summary of average non-accidental mortality, temperature and other study-specific variables across the 20 cities is reported in Table I.

**Table I:** Mean, range and specific percentiles for study-level variables in 20 US cities, 1987–2000

	Mean	Min	25th	75th	Max
Average non-accidental mortality counts	22.3	4.5	9.2	23.3	115.4
Mean temperature (°C)	11.0	8.4	9.9	12.0	14.3
Minimum temperature (°C)	−24.3	−28.6	−25.0	−22.8	−20.8
Maximum temperature (°C)	31.8	29.4	31.0	32.4	34.4
Latitude (degree north)	40.8	38.0	39.6	42.5	43.2
Population size ( × 100 000)	9.7	1.7	4.1	9.8	53.8
Urbanized population (%)	94.0	73.9	90.7	99.1	100.0
Population with high education (%)	82.8	71.3	81.6	85.7	92.2
Population living in poverty (%)	12.6	8.9	11.2	13.2	24.6
Population unemployed (%)	6.0	3.8	5.0	6.5	11.3

### 2.3. Modelling strategies

The analysis is performed in two stages, as described in the framework summarized previously. In the first stage, a regression model is applied to individual data for each of the 20 cities included in the analysis in order to derive study-specific estimates of the non-linear exposure–response association, as described in detail in Section 3. The estimated study-specific relationship is entirely defined by the parameters of the function chosen for representing the association, namely, a set of regression coefficients. These coefficients are then used as outcomes for the multivariate meta-analytical model in the second stage, following the methodology described in Section 4. As in conventional univariate meta-analysis, the main aim is to derive a set of regression coefficients defining an average exposure–response association across the studies. In a second step, we aim to test and quantify the amount of heterogeneity and to assess the extent to which this heterogeneity is related to study-specific characteristics, such as those listed in Table I, through multivariate meta-regression.

## 3. First-stage model

We suppose that *N*_*i*_ observations, indexed by *t* = 1, …, *N*_*i*_, have been collected in study *i* for each of the *i* = 1, …, *m* studies. Specifically, the data include observations on a response variable *Y*_*ti*_, an exposure of interest *z*_*ti*_, and an optional set of *J* confounding variables *c*_*tji*_, with *j* = 1, …, *J*. The aim of the first-stage analysis is to define and estimate the association between *Y*_*ti*_ and *z*_*ti*_, while controlling for *c*_*tji*_. This association is specified through a function *s*, entirely defined in each study by the *k*-length vector of parameters ***θ***_*i*_.

Alternative options are available regarding the choice of the function *s*, including different types of splines [19], polynomials or fractional polynomials [20], step functions, segmented regression [21] and other multi-parameter functions. Also, the function *s* may describe multi-parameter associations other than non-linearity, although the latter is likely to represent the principal application. Similarly, the estimation of the regression parameters is usually performed through a regression model, such as generalized linear or Cox proportional hazard models, whose specification is dependent on the study design and type of outcome. Whatever the modelling choices for the function *s* or type of regression, the purpose is to obtain for each study the *k*-length vector of estimated parameters 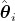 and accompanying *k* × *k* estimated (co)variance matrix **S**_*i*_. These are assumed to be derived from an estimator for the unknown parameters ***θ***_*i*_, which define the true study-specific associations. The second-stage multivariate meta-analytical framework, described in Section 4, is general and not dependent on choices about the first-stage model and parameterization.

### 3.1. First-stage model used in our example

In our example, the first-stage analysis is carried out using a generalized linear model with quasi-Poisson family for overdispersed data, following a standard analytical approach for time-series environmental data [22, 15]. The general algebraical definition is given by the following:


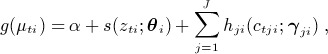
(1)

where *g* is a log link function of the expectation *μ*_*ti*_ ≡ *E*(*Y*_*ti*_), with *Y*_*ti*_ as the series of 5114 non-accidental daily mortality counts. The exposure *z*_*ti*_ corresponds to outdoor mean daily temperature over lag 0–5, computed as the moving average on day *t* and on the previous 5 days, in order to account for lagged effects. Here, the exposure–response function *s* is chosen as a quadratic B-spline, entirely defined by *k* − 2 internal knots and 2 boundary knots, where *k* corresponds to the dimension of the spline basis and the number of parameters. This is equal to the degrees of freedom (*df*) spent in the first-stage model to estimate the relationship. Number and location of knots are chosen by the selection method detailed in Section 3.3.

The model also includes two potential confounding variables *c*_*tji*_, included a priori, represented by elapsed time and day of the week, modelled through the functions *h*_*ji*_ and related parameters ***γ***_*ji*_. Elapsed time is used to control for seasonal and long-term trends and specified through a natural cubic B-spline with 7 *df* per year. Day of the week is modelled with six indicator variables through a dummy parameterization.

### 3.2. Definition of the exposure–response function

In Equation (1), while defining the first-stage regression model, we intentionally omitted the index *i* for the function *s* to indicate that the same function needs to be applied in all the study-specific models. This requirement assures that the estimated coefficients 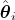 retain the same interpretation in all the *m* studies, so that their meta-analysis can provide meaningful and interpretable results. In our example, this condition is met by placing the internal and boundary knots at the same temperatures in all the *m* studies. Functions for non-linear associations are usually centred on a specific value, so that the results may be interpreted as the effect of the exposure versus a reference [23]. The choice of the centering value depends on interpretational issues and does not affect the fit of the model in (1).

### 3.3. Control for confounders and model selection

The specification of the study-specific models in (1) can follow two different approaches regarding the control of confounders. In this example, the confounder model, although involving several terms, is conceptually simple, and, consistently with previous time-series environmental studies on the same dataset [22], an identical set of pre-specified predictors is included in all studies. An alternative approach is based on study-specific confounder models. The choice depends, among other issues, on the research setting and the quality of the data. Whatever the strategy adopted to control for confounding in the first stage, the aim is to obtain a valid estimate of the study-specific parameters ***θ***_*i*_, and this choice does not affect the development of the second-stage multivariate meta-analysis.

Different selection methods are available for the definition of the function *s*. In this illustration, the choice reduces to the identification of the optimal number and location of knots for the quadratic B-spline. In the context of time-series environmental studies, several criteria have been proposed, although consensus as to an optimal approach has not been reached yet. Here we rely on the Q-AIC, a modification of the Akaike information criterion (AIC) for quasi-likelihood models [24]. We first define a list of model candidates, specified by an increasing number of internal knots placed at temperatures corresponding to optimal average percentiles across cities [25], Section 2.4. Boundary knots are placed at temperatures corresponding to the average minimum and maximum temperature. The selected model has the minimum value of the sum of the Q-AIC in all the 20 studies. As with other issues discussed previously concerning the first-stage model, different selection criteria may be preferred in different settings.

### 3.4. Absolute and relative scales

The meta-analysis of exposure–response relationships requires that the exposure is measured on the same scale in all the studies. Also, in the presence of different exposure ranges, common knots placement may leave some parameters inestimable in some studies. The meta-analysis of their parameters therefore presents additional complications.

The modelling strategy described previously defines the exposure on an *absolute* scale (°C). The function *s*, common to all studies, is coherently defined with knots placed at the same absolute exposure values, so that the estimated coefficients have the same interpretation across studies. Their synthesis through meta-analysis is therefore meaningful. In our example, selected cities have largely overlapping temperature ranges, as showed in Table I, and the specification of common interior and boundary knots does not raise important issues. However, in analyses including studies with more varied temperature ranges, such as of all the 108 US cities, the definition of common knots would be problematic or impractical.

An alternative is to adopt a *relative* scale, standardizing the study-specific distributions by transforming temperatures to the related study-specific percentiles. This method allows the comparison of studies showing even non-overlapping ranges. The standardization is carried out by selecting the internal knots at the same percentiles in all the studies, which indeed correspond to different absolute temperatures. The interpretation of the estimated coefficients changes, and the comparison must be made on the relative scale of percentiles, as illustrated while commenting on the results in Section 5.4.

## 4. Multivariate meta-analysis

The study-specific estimates obtained from the first-stage model are then combined through multivariate meta-analysis. The theoretical arguments that underpin the definition of this modelling framework closely follow the simple univariate model, recently re-evaluated in detail [26]. A thorough overview on the multivariate extension has been provided in a recent paper [11]. However, in contrast to the original setting of randomized controlled trials, in the current multi-parameter setting it is not necessary that the parameters are individually interpretable, and the association is instead characterized through their joint distribution. This specific feature constitutes the object of the re-assessment we provide in this section, often referring to the example described in Section 2.

### 4.1. The model

The framework we use is nested within that of the multivariate normal linear mixed model and so follows well-developed lines [27]. It will be presented in the specific context of multi-parameter associations. As anticipated previously, we assume that a first-stage model has been fitted to the data from each of the *i* = 1, …, *m* studies, obtaining *k*-length vectors of regression coefficients 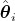, and associated *k* × *k* estimated (co)variance matrices **S**_*i*_. These regression coefficients are then used as outcomes for the second stage and are termed from here on as outcome parameters in order to distinguish them from the coefficients of the second-stage meta-analytic model.

Following Jackson and colleagues [11], a model for random-effect multivariate meta-analysis can be written as follows:


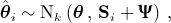
(2)

with **S**_*i*_ + ***Ψ*** = ***Σ***_*i*_. The marginal model in (2) has independent within-study and between-study components. In the within-study component, the estimated 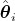 is assumed to be sampled with error from N_*k*_(***θ***_*i*_,**S**_*i*_), a multivariate normal distribution of dimension *k*, where ***θ***_*i*_ is the vector of true unknown outcome parameters for study *i*. In the between-study component, ***θ***_*i*_ is assumed sampled from N_*k*_(***θ***,***Ψ***), where ***Ψ*** is the unknown between-study (co)variance matrix. Here ***θ*** can be interpreted as the population-average outcome parameters, namely the coefficients of the function *s* defining the average exposure–response association. Model (2) can be extended to multivariate meta-regression, where the *k* outcomes are modelled in terms of study-level variables defining a set of *p* meta-predictors **x**_*i*_ = [*x*_1*i*_,*x*_2*i*_, …, *x*_*pi*_]^*T*^ associated with the *i*th study, where usually *x*_1*i*_ = 1 specifies intercept terms. Algebraically,


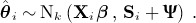
(3)

The *k* × *kp* block-diagonal matrix **X**_*i*_, of rank *kp*, is derived by the Kronecker product between an identity matrix **I**_(*k*)_ of dimension *k* and the vector **x**_*i*_, following


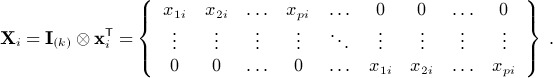
(4)

The *kp*-dimensional coefficient vector ***β*** defines the association of the *k* outcomes with *p* meta-predictors. They commonly include *k* intercepts with a similar interpretation of ***θ*** in (2) but related to the population average of studies characterized by a zero value of meta-predictors. The other *k*(*p* − 1) coefficients in ***β*** express how the outcome parameters change in respect to the meta-predictors. The problem can also be re-expressed in the form of a conventional linear mixed model, defining random effects ***u***_*i*_ ∼ N_*k*_(**0**, ***Ψ***), which represent study-specific deviations from the average. The model in (3) is then written as follows:


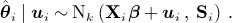
(5)

The matrix ***Ψ*** is completely defined by a set of parameters ***ξ***, dependent on the chosen structure and parameterization. If no a priori structure is assumed, *k*(*k* + 1) ∕ 2 terms are needed. Optionally, under the assumption that each outcome parameter is explained only in terms of a subset of the *p* variables, the related columns of **X** and entries of ***β*** can be excluded, defining different linear predictors. Fixed-effect meta-analytic models presuppose that no heterogeneity exists in the distribution of the outcome parameters and that the random variability is explained only by sampling error, assuming ***Σ***_*i*_ ≡ **S**_*i*_. As for the univariate case, estimation procedures treat **S** as known. From here on, the model in (2) for multivariate meta-analysis will be considered a special case of (3)–(5) where **X** ≡ **I** and ***β*** ≡ ***θ***. The unknown parameters are therefore ***β*** and, for random-effect meta-analytic models, ***ξ***.

### 4.2. Estimation

Different estimation methods have been proposed for random-effect multivariate meta-analysis: likelihood-based methods 9, 28, estimating equations [29], variants of iterative generalized least squares [8, 30], Bayesian approaches [31] and multivariate extensions of the method of moments [32]. Here we will concentrate on maximum likelihood (ML) and restricted maximum likelihood (REML), following an extensive literature within the framework of linear mixed models [27, 33, 34]. These methods are implemented in the R package mvmeta and applied to perform the analysis in Section 5.

The marginal log-likelihood function *ℓ*(***β***,***ξ***) for model (5) may be written as follows [27]:


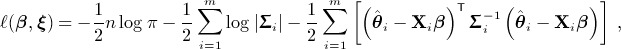
(6)

with ***Σ***_*i*_(***ξ***) written here as ***Σ***_*i*_ for ease of notation and *n* as the total number of observations (usually equal to *km* where there are no missing values). Assuming that ***ξ***, and therefore ***Ψ*** and ***Σ***, are known, the ML estimates for ***β*** and its (co)variance matrix 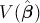 conditional on ***ξ*** are obtained by maximizing (6). In this case, closed-form equations are given by generalized least squares estimators:


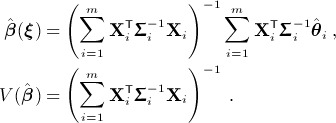
(7)

When ***Ψ*** is not known, the joint likelihood function in (6) needs to be maximized with respect to both ***β*** and ***ξ***, and iterative methods are required. However, the ML estimator of the (co)variance parameters ***ξ*** is usually biased downward, as it does not account for the loss of degrees of freedom from the estimation of ***β***. An alternative estimator can be obtained from the maximization of the log-likelihood function on the basis of a set of *n* − *q* linearly independent error contrasts, with *q* as the number of fixed effects coefficients in ***β***. This restricted log-likelihood (REML) function *ℓ*_*R*_(***ξ***), not dependent on ***β***, may be conveniently expressed as follows [27, 33]:


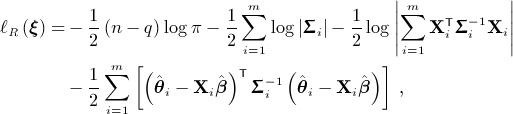
(8)

where 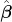 is defined in (7) and ***Σ***_*i*_ identifying ***Σ***_*i*_(***ξ***) as above.

The ML estimates of ***β*** in fixed-effect meta-analysis are simply obtained by (7), given that, as discussed in Section 4.1, ***Σ***_*i*_ equals **S**_*i*_ and is therefore completely known. The ML and REML estimates in random-effect models can be instead obtained through Newton-type iterative algorithms. For computational purposes, the objective functions in (6) and (8) are both expressed with respect to ***ξ*** only, and maximization of *ℓ*(***ξ***) and *ℓ*_*R*_(***ξ***) can be achieved by plugging in at each iteration the conditional estimate of 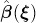 in (7) using the current estimate of ***ξ***, until convergence. Additional information on the estimation algorithms used here are provided in the Supplementary Web Appendix.

### 4.3. Hypothesis testing and model comparison

We can separate inferences about the parameters in models (2) and (3)–(5) into those about the fixed effects ***β***, which will typically be of prime interest, and the between-study (co)variance matrix ***Ψ***. Inferential procedures, again, follow the theory of linear mixed models [[27], Chap. 6].

Regarding fixed effects, under the marginal model and assuming ***ξ*** as known, 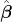 follows a multivariate normal distribution with mean and (co)variance matrix given in (7). By replacing ***ξ*** by its ML or REML estimator, the corresponding entries of the resulting 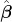 and 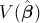 may be used for obtaining significance tests or confidence intervals for single coefficients. However, in this specific context, the inference is focused on the set of coefficients defining the association of a specific study-level variable with the joint distribution of the outcome parameters. Multivariate extension of Wald or likelihood ratio (LR) tests are easily derived for this purpose. As usual for REML models, general likelihood theory does hold for LR tests only when comparing models with identical fixed effects structures [33]. Suitable adjustments for the underestimation of (co)variance error matrices due to the uncertainty in the estimation of ***Ψ*** are available [35, 36] although not yet been implemented in mvmeta.

For random effects, the focus is on comparing models involving different choices about the structure of the between-study (co)variance matrix. In this setting, an interesting hypothesis to test is ***Ψ*** = **0**, namely that no heterogeneity between studies exists beyond that explained by sampling variability. Similarly, a LR test between nested models may be performed, which is appropriate in REML models as well, given the identical fixed effects structures. Note, however, that for alternative hypotheses that constrain (co)variance matrices to be positive-definite (Supplementary Web Appendix), the null value lies on a boundary of the parameter space. Under these conditions, the conventional null asymptotic 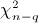 distribution does not hold, and some adjustment has been proposed [37]. A test for the same null hypothesis and distribution has also been developed as the multivariate extension of the Cochran *Q* test for (residual) heterogeneity [7, 29]. The test is based on the statistic:


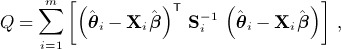
(9)

where 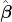 are estimated by the correspondent fixed-effect model. An extension of this heterogeneity test for a subset of ***β*** has also been proposed [29].

In addition, in this meta-analytical setting, the quantification of the heterogeneity among studies, or the residual amount beyond that explained by specific covariates, is also of interest. Indices of heterogeneity analogous to the univariate case may be easily derived, such as the *H*^2^ and *I*^2^ statistics [38]. These measures are interpreted as the relative excess in heterogeneity (above that explained by sampling error) and the proportion of total variation attributable to heterogeneity, respectively. Although recently criticized for being dependent on precision of the estimates from the first-stage model [39], these statistics provide simple summaries on the extent of heterogeneity. These measures may be produced for the joint multivariate distribution of the *k* parameters and interpreted as the amount of heterogeneity in the exposure–response relationships. In this case, from (9), we derive the following:


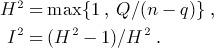
(10)

The same statistics may also be defined for single-outcome parameters [40] by simply computing the related contribution to the quantity *Q* and *df* = *n* − *q* while applying (10). However, as previously discussed, the interpretation of these measures for single parameter of a spline function is not very informative.

More broadly, non-nested models may be compared using fit statistics, in particular Akaike information criterion, 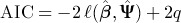, and Bayesian information criterion, 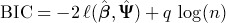, where 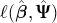 is the maximum log-likelihood. These statistics may also be used with REML models, with the additional requirement that fixed effects structure be held constant.

### 4.4. Prediction

In the context of multi-parameter associations, the general tests and fit criteria described previously, although important, are usually insufficient for interpretation. Coefficients in ***β*** refer to single-outcome parameters that are rarely interpretable on their own, and the tests only offer a statistical belief on *whether* the multivariate distribution of outcome parameters depends on study-level covariates. However, these procedures fail to inform on *how* the latter modifies the former.

In the current setting, prediction represents an important tool to extend the inference from multivariate meta-regression models, offering a method to link specific values of study-level variables with outcome parameters expectations. Given a set of meta-predictor values **x**_0_, the model-predicted mean 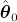 and (co)variance matrix 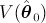 are obtained by the following:


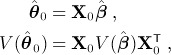
(11)

with **X**_0_ computed from **x**_0_ following (4). The equations in (11) may be used to predict specific parameters 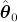, which are used to define the average exposure–response curve predicted for the set of meta-predictor values **x**_0_. The same equations may be used to predict the association in a new study characterized by a specific set of study-level variables by simply increasing the uncertainty in the estimates by adding 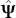 to 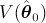 in (11) [41].

In addition, the assumptions outlined in Section 4.1 regarding the random-effect multivariate distribution may be exploited to extend the inference regarding study-specific outcome parameters ***θ***_*i*_ estimated in the first-stage model, computing the (asymptotic) best linear unbiased prediction (BLUP) [[27], Section 7.4]. The predicted 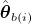 and associated asymptotic (co)variance matrix 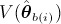 are as follows:


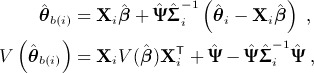
(12)

for 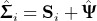. The BLUP equations in (12) represent the sum of two components: the predicted averaged outcome parameters in (11) and study-specific deviations, predicted as random effects ***u***_*i*_ in (5). Exposure–response associations predicted with BLUP in (12) represent a trade-off between city-specific and average estimates, with weights inversely proportional to the two components ***Ψ*** and **S**_*i*_ of the total variability ***Σ***_*i*_, respectively. It is noteworthy, in this multivariate setting, that the BLUP estimates of missing parameters from the first stage exploit the information about the other study-specific parameters obtained through the between-study (co)variance matrix 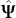 and may be therefore different from predicted values from (11).

## 5. Results

The first-stage generalized linear model in (1) is fitted to the data from each city, considered as independent studies. The selection procedure indicates a preference for a quadratic B-spline with 6 *df*, with 4 interior knots at − 4.7°C, 6.0°C, 16.7°C and 24.8°C, corresponding to the 5th, 35th, 65th and 95th average distribution percentiles across studies. The boundary knots are set to the average minimum and maximum, ranging from − 18.3°C to 29.2°C. All the spline basis variables are centred at 20°C. The vectors 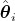 and (co)variance matrices **S**_*i*_ for the six parameters are then pooled across studies in multivariate meta-analysis and meta-regression. All the models are fitted through ML. Given the illustrative purpose of this example, in the description of the results we favour methodological matters over subject-specific details. All the results described in this section may be reproduced using the R code available in the Supplementary Web Appendix.‡

### 5.1. Pooled exposure–response relationship

**Figure 2:**
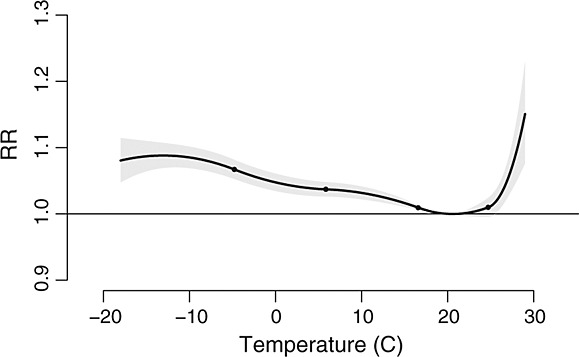
Pooled exposure–response relationship in relative risk between temperature and non-accidental mortality in 20 US cities, 1987–2000. The grey area represents 95% confidence intervals. Dots indicate knots location. Reference at 20°C.

The main result of multivariate meta-analysis, applying the model in (2), consists of an estimate 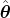 of the outcome parameters ***θ***, representing the population-average coefficients of the quadratic B-spline. These are used to compute the average exposure–response relationship depicted in Figure 2, together with confidence intervals obtained through 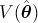, over the average exposure range. As expected, the plot indicates an increase in risk for non-accidental mortality for both high and low temperatures, with a point of minimum mortality at about 20.6°C. The risk increases approximately linearly in the cold tail but shows a steep non-linear increase at high temperatures. The model estimates a relative risk (RR) of 1.085 (95%CI: 1.069–1.102) and 1.150 (95%CI: 1.076–1.230) for temperatures of − 10°C and 29°C versus 20°C, respectively. The multivariate Cochran *Q* test and *I*^2^ obtained from (9)–(10) are reported in Table II. These statistics reveal a significant heterogeneity across studies, where 70.2% of the variability in the multivariate outcome 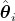, and therefore in the derived exposure–response association, is attributed to true between-study differences.

**Table II:** Cochran *Q* test, *I*^2^ and information criteria for second-stage multivariate models based on an absolute or relative scale

	Cochran *Q* test			*I*^2^	Information criteria		LR test			Wald test		
					
	*Q*	*df*	*p*	%	AIC	BIC	Stat	*df*	*p*	Stat	*df*	*p*
Absolute scale												
Intercept only	382.8	114	0.000	70.2	−322.8	−247.5						
Latitude	186.0	108	0.000	41.9	−326.5	−234.5	15.7	6	0.015	30.9	6	0.000
Urban population	342.8	108	0.000	68.5	−317.9	−226.0	7.1	6	0.309	8.7	6	0.191
High education	376.4	108	0.000	71.3	−316.1	−224.1	5.3	6	0.506	8.2	6	0.227
Living in poverty	365.8	108	0.000	70.5	−314.9	−222.9	4.1	6	0.661	5.4	6	0.493
Relative scale												
Intercept only	304.5	114	0.000	62.6	−344.2	−268.9						
Latitude	258.7	108	0.000	58.3	−338.4	−246.4	6.3	6	0.393	7.7	6	0.265

Likelihood ratio (LR) and Wald tests, also included, refer to the association with meta-predictors in meta-regression models.

### 5.2. Best linear unbiased prediction

**Figure 3:**
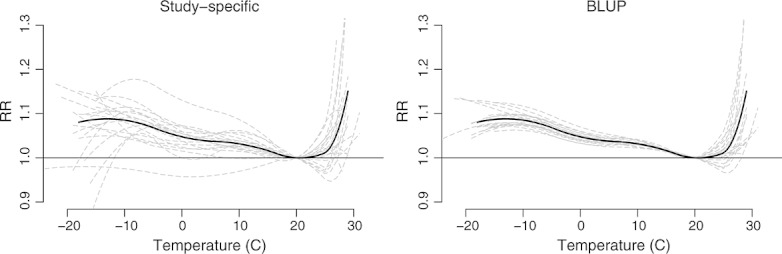
First-stage (left) and best linear unbiased predicted (right) estimates of the exposure–response relationships in relative risk between temperature and non-accidental mortality in 20 US cities, 1987–2000. The continuous bold black line represents the population-average curve, whereas the long-dashed grey lines are the study-specific estimates. Reference at 20°C.

As discussed in Section 4.4, the assumptions about the between-city variability, namely the distribution of the random effects in (5), can be used to extend the inference regarding study-specific estimates. Variability around the average is due to both heterogeneity between cities and uncertainty in the first-stage model. Applying (12), we derived the BLUP estimates 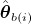 of the unknown city-specific parameters ***θ***_*i*_ of the quadratic spline, representing a shrunk version of the estimated 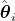 towards the population average 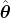. Figure 3 illustrates the associated exposure–response curves: the left panel shows the original estimates from the first-stage models in the 20 studies, whereas the related BLUP relationships are displayed in the right panel. The shrinkage effect is stronger in the cold extreme, possibly because of the higher uncertainty in the within-study estimates.

**Figure 4:**
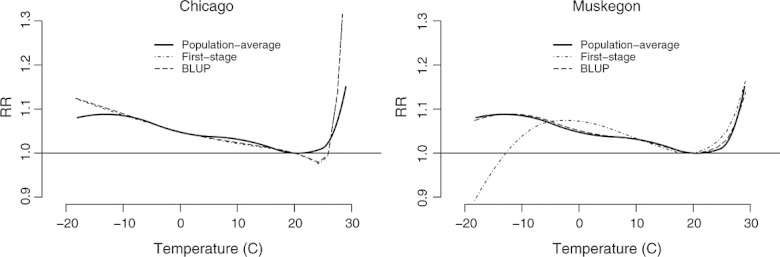
Population-average (continuous bold line), first-stage (dash–dot line) and best linear unbiased predicted (dash line) exposure–response relationships in relative risk between temperature and non-accidental mortality in two US cities, 1987–2000. The figure illustrates the largest (Chicago, left) and smallest (Muskegon, right) studies included in the analysis. Reference at 20°C.

Given the large difference in population size shown in Table I and in the related within-study precision, the shrinkage effect is expected to vary considerably across studies. Figure 4 shows the first-stage and BLUP temperature–mortality relationships in two studies, together with the population average as depicted in Figure 2. As expected, the BLUP estimate is closer to the original curve in Chicago, the largest city characterized by a high number of daily deaths (above 5 million inhabitants, 115 deaths on average per day). By contrast, the study in the smallest city of Muskegon (170 000 inhabitants, five deaths per day) produces very imprecise estimates (confidence intervals not shown), in particular for cold, which are heavily shrunk toward the population average.

### 5.3. Multivariate meta-regression

Part of the large heterogeneity in temperature–mortality curves between studies, displayed in Figure 3, may be explained by different patterns of study-level factors that modify the association. Multivariate meta-regression models in (3)–(5), including meta-predictors, are applied to extend the analysis and partly characterize such differences. From a fitted model, it is possible to compute the outcome parameters 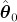 predicted for a set of meta-predictor values **x_0_** using (11). As discussed in Section 4.4, these parameters are interpreted as the average coefficients of the function *s* for the subset of studies from the hypothetical population that are characterized by **x** = **x**_0_. A test on the significance of the multivariate association between the outcome parameters and each study-level variable is carried out by applying a Wald test on the related subset of coefficients ***β***. Alternatively, an LR test or information criteria such as AIC and BIC can be used to compare the models with and without specific meta-predictors.

**Figure 5:**
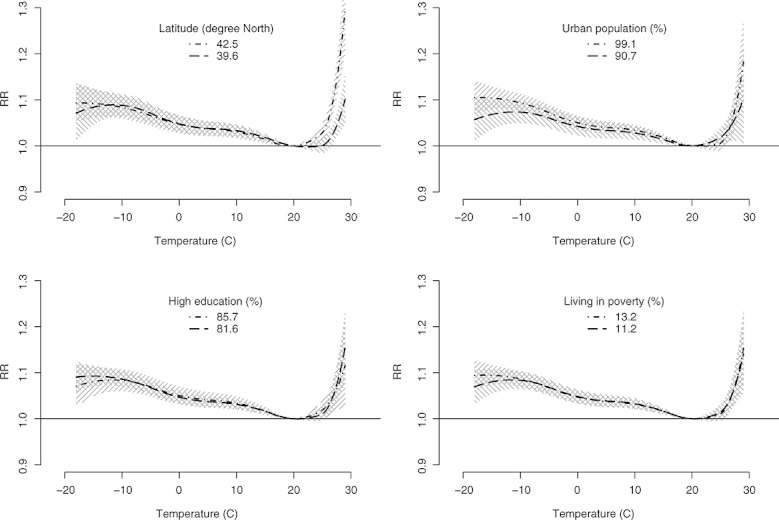
Predicted exposure–response relationships in relative risk between temperature and non-accidental mortality in 20 US cities, 1987–2000. The figure illustrates the predicted curves from meta-regression for the 25th (dash line) and 75th (dash–dot line) percentiles of latitude (top left), percentage of population living in urban settings (top right), percentage of population with high education (bottom left) and percentage of population living in poverty (bottom right). The shaded areas represent 95% confidence intervals. Reference at 20°.

As an illustration, univariable multivariate meta-regression models, each including a single meta-predictor, are specified for four study-level variables, namely latitude, percentage of population living in urban settings, percentage of population with high education (high school or higher) and percentage of population living in poverty. The exposure–response associations are predicted for the values of the approximate 25th and 75th percentiles of the study-level variables (reported in Table I) and shown in Figure 5. The top-left panel illustrates the two predicted curves for latitude. The effect of hot temperature, on average, is higher in studies for northern cities, with a steeper raise in risk: the RR at 29°C versus 20°C increases from 1.101 (95%CI: 1.049–1.156) for the 25th percentile of latitude to 1.297 (95%CI: 1.205–1.396) for the 75th percentile, although there is also a suggestion of a lower point of minimum mortality when increasing latitude. No substantial effect modification is suggested for cold temperatures. The importance of the overall association with the study-level variable can be assessed by the comparison of the AIC and BIC criteria, reported in Table II, with the corresponding model with no meta-predictor in Figure 2. The lower AIC, from −322.8 to −326.5, suggests a better fit of the model with the predictor, whereas the BIC, highly penalized by the number of observations in the model, shows a preference for the more parsimonious model. The Wald and LR tests, also reported in Table II, are both specified with 6 *df*, corresponding to the six additional coefficients used to model the linear effects of latitude on each outcome parameters. The tests are both significant at the standard 5% level, although the *p*-values differ somewhat. Latitude seems to explain a substantial part of heterogeneity between studies, with an overall *I*^2^ of 41.9% compared with the 70.2% of the model with no predictor. However, the test for the residual amount of heterogeneity is still significant.

The other three panels in Figure 5 show the association with the other study-level variables. The percentage of population living in urban settings seems to increase the effect of both cold and hot temperatures, although fit statistics and tests in Table II suggest that this effect modification is not significant. Also, the decrease in heterogeneity is negligible. There is no evidence that percentage of population with higher education or living in poverty modify the temperature–mortality association, as shown by the two identical curves predicted for their 25th and 75th percentiles in the bottom panels of Figure 5 and related tests and fit statistics in Table II.

The extension to multivariable multivariate meta-regression is straightforward: tests and statistics are defined in exactly the same way, and predicted effects as those showed in Figure 5, controlled for the effect of other study-level variables, may be computed in a similar way. The interpretation, as with standard multivariable models, refers to the effect of a meta-predictor for constant values of the other ones. Standard model selection procedures for regression models may be applied to choose the set of meta-predictor to be included. An example is included in the code provided in the Supplementary Web Appendix.

### 5.4. Adopting a relative scale

**Figure 6:**
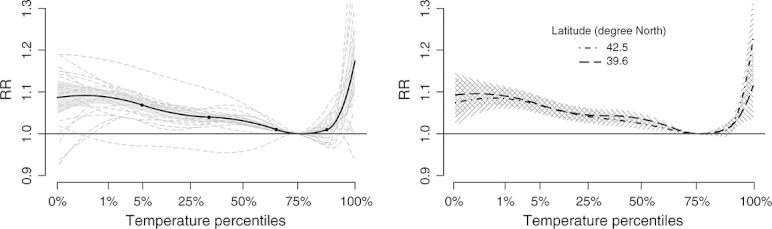
Pooled and predicted exposure–response relationships in relative risk between relative temperature (percentiles) and non-accidental mortality in 20 US cities, 1987–2000. The *x*-axis is scaled so that percentiles match those of the average temperature distribution of all the cities. The figure illustrates the population-average curve from meta-analysis and study-specific estimates (left panel, dots indicate knots location) and the predicted curves from meta-regression for the 25th (dash line) and 75th (dash–dot line) percentiles of latitude (right panel). Reference at the 75th percentile of temperature.

As shown in Table I and in Figure 3, the exposure ranges are slightly different in each study. In this specific example, range variation was too small to cause problems, but it is likely to do so when exposure ranges differ more substantially. An alternative is to adopt a relative scale based on percentiles, as described in Section 3.4. As an illustration, we repeat the analysis placing the knots at the 5th, 35th, 65th and 95th percentiles of study-specific temperature distribution and centering on the 75th percentile. The predicted exposure–response relationship is displayed in Figure 6. To aid interpretation in this standardized scale, the *x*-axis is scaled so that percentiles match those of the average temperature distribution across all the cities.

Not surprisingly, given the similar temperature ranges, the shape of the pooled association shown in the left panel of Figure 6 is very close to that predicted in absolute scale in Figure 2. However, the results from the two approaches are not easily comparable because of the different interpretation of the results. The exposure–response relationship predicted in an absolute scale informs about the average RR between specific temperatures, for example 30°C versus 20°C. By contrast, the curve predicted in relative scale measures the RR of relative temperatures, for example 95th versus 75th percentiles, based on study-specific temperature distributions. The right panel of Figure 6 illustrates the results from the meta-regression model including latitude. Although the risk of hot temperatures is still higher in northern cities, the difference is smaller if compared with the related plot predicted in absolute scale in Figure 5, top-left panel, and no longer significant, as shown in Table II. Also, the point of minimum mortality is similar in cities at different latitudes, approximately at the 76th percentile. This may be explained by a partial adaptation of populations living in different cities to their own climate.

### 5.5. Additional analysis

The selection procedure regarding the function *s* described in Section 3.3 indicates a preference for a spline with 4 knots. As a sensitivity analysis, the results in Section 5.1 are compared with those obtained using a spline with 3 knots, placed at − 1.8°C, 11.3°C and 23.3°C, corresponding to the 10th, 50th and 90th average distribution percentiles across studies. The exposure–response curve is very similar to that showed in Figure 2 (result not shown), suggesting that the choice of number and position of knots is not critical in this case.

All the models presented previously have been estimated through ML. The choice of this estimator allows the comparison of models with different fixed effects structures through LR test and information criteria, as described in Sections 4.2–4.3. The estimation can be potentially improved through the use of REML, which accounts for some additional uncertainty in the estimation of the between-study (co)variance matrix ***Ψ*** not accounted by ML estimates. However, even with this relatively limited number of studies, standard errors of coefficients for REML models only increase in the range of 1–2%, and the two predicted exposure–response curves and confidence intervals are almost identical (Supplementary Web Appendix).

As previously pointed out, estimation difficulties may arise in this complex modelling framework, particularly regarding between-study correlations close to the boundary of their parameter space of − 1 or 1 [11]. The procedure implemented in mvmeta, based on Cholesky decomposition to assure positive-definiteness, seems to perform well under several modelling choices using these data, and estimation problems have not occurred.

In meta-regression models presented in Section 5.3, 120 correlated outcomes from 20 studies are used to estimate 12 coefficients and 21 (co)variance parameters. These sophisticated models require several assumptions that are commonly hard to verify, such as the multivariate normality of the random effects ***u***_*i*_ in (5). The results are therefore expected to be potentially sensitive to model mis-specifications. In particular, we have noticed some discrepancies between the Wald and LR tests in meta-regression models in Figure 5, probably because of different assumptions of the two tests. In a preliminary assessment, the quadratic approximation to the likelihood surface of the Wald test seems to perform poorly in badly conditioned models, for example meta-regression in the presence of outliers. Fitted models may be checked with appropriate regression diagnostics, such as residuals and influence measures already available for univariate meta-analysis [42], which still need to be developed in the multivariate meta-analytical techniques.

## 6. Further considerations

As mentioned earlier, the methodology of multivariate meta-analysis has been largely developed in the context of randomized controlled trials to pool estimates of multiple outcomes. Moreover, as shown in Section 4, the statistical framework may be placed within that of the general linear mixed model, although with particular characteristics. Nevertheless, specific issues arise when this methodology is applied to multi-parameter associations in two-stage analyses. Here we provide some comments on these aspects, highlighting advantages and limitations and directions for future research.

*Advantages of multi-parameter synthesis*. As anticipated in Section 1, the application of multivariate meta-analysis extends the standard two-stage design, where the data on the associations of interest are usually summarized in the estimate of a single parameter. For complex associations, the traditional approach may be too limited to characterize the phenomenon under study. Referring to the examples illustrated in Section 5, a standard analysis can be based on the pooling of single estimated effects at specific percentiles for temperature [18], or on simplification of the relationship through linear-threshold parameterization [17]. The estimate of the truly non-linear exposure–response relationship is more informative and flexible and may be preferred for the investigation of complex associations. This approach may be more broadly described in the context of multi-parameter evidence synthesis [43, 44].

*Dealing with complexity*. The two-stage approach discussed previously provides tools for the analysis of complex associations. In the first step, the estimate is controlled for potential confounders while reducing the relationship to a limited number of parameters of a chosen function, which are subsequently used as outcomes for the second-stage meta-analytic model. The amount of complexity retained in the first stage represents a trade-off between the synthesis of simple approaches and details offered by sophisticated models. Ideally, this balance should be fine-tuned only to the purpose of the analysis. However, in practice, concrete problems such as mathematical and statistical properties of the function or the number of parameters need to be taken into account, as discussed in the following paragraphs. Moreover, this complex framework involves important modelling assumptions and is particularly sensitive to model mis-specification, as pointed out in Section 5.5. Further research is needed to develop this approach as a standard analytical tool.

*Dimensionality and sample size*. Using this type of approach, the dimension *k* of the parameter vector that can be accommodated in multivariate meta-analysis is of course limited, as *kp* fixed effects coefficients and *k*(*k* + 1) ∕ 2 (co)variance parameters need to be estimated. Guidance on the sample size (i.e. number of studies) for multivariate meta-analytical models is not yet available, and additional research is required to better characterize this complex modelling framework [11]. A possible solution to reduce the number of parameters is to structure the between-study (co)variance matrix ***Ψ***, for example imposing compound-symmetry, diagonal or autoregressive forms. Robust estimation to account for wrong correlation structures has been proposed for meta-regression of correlated outcomes [45], although further research is needed for this approach.

*The two-stage design*. In the setting of randomized controlled trials, the two-stage approach is often compared with the so-called one-stage IPD analysis, usually performed through a single multilevel model. Although the latter has been advocated as more efficient and less prone to bias if compared with the meta-analysis of published studies [46, 47], the two-stage alternative has been proved as competitive when applied through IPD analysis [48, 49]. In addition, a single multilevel development is not always feasible or advisable, especially in the presence of many individual-level covariates, which would require the definition of intricate study-specific dependencies. In our example, 7 × 14 = 98 parameters are used in the first stage to model within-study seasonal and long-term trends. In the two-stage framework, parameters not related to the association of interest are treated as nuisance terms in the first-stage model, offering computational efficiency and flexibility regarding model specification and assumptions.

*Analysis of published studies*. Although the modelling framework proposed here is focused on two-stage analysis of complete study-specific datasets, most of the original development of meta-analysis is based on the combination of estimates from published results. This also applies to the multivariate extension, as described in Section 7. The meta-analysis of published studies poses additional problems. First, the outcome parameters defining the association in each study may not be comparable, for instance if estimated from different functions. Referring to the application in which non-linear dependencies are modelled, exposure categories may be defined with different cut-offs or spline functions with different knots. Solutions have been previously proposed to retrieve estimates of comparable outcome parameters from available study-specific information, as discussed in Section 7. Another issue is that correlation between estimated outcome parameters are rarely reported. Methods to deal with missing correlations have been developed [50, 51], although this issue needs to be explored further, especially for multi-parameter associations.

*Definition of the function s*. As discussed previously, the choice of the function is primarily dictated by the purpose of the analysis and the amount of complexity needed to represent the association. Also, the exact specification of the function may be the result of a data-led selection procedure. A main limitation of the proposed approach is the requirement to specify the same function in all the studies, a constraint that could have a strong influence on study-specific estimates. In particular, the selected function might not fit the data well in some studies, while producing the best fit overall. As showed in Section 5.5, the sensitivity of the results to modelling choices and selection should always be inspected.

*Exposure ranges and absolute or relative scale*. In our illustrative example, the 20 studies showed similar exposure ranges, and the meta-analysis was first performed adopting an absolute scale. However, as already mentioned in Section 3.4, this approach is not feasible to pool studies characterized by more varied exposures ranges. In such an analysis, the definition of a common function *s* is not straightforward. In the specific example illustrated here, the selection of common knots may result in some parameters of the function being inestimable. The interpretation of the other city-specific outcome parameters would change, and the meta-analysis, although feasible in the presence of missing outcomes, may produce results of uncertain meaning. The analysis using a relative scale represents an interesting and feasible alternative, although it involves a different interpretation of the results, as described in Section 5.4. In temperature–mortality associations, the relative scale accounts for adaptation of populations to their own climate, but this approach may not provide meaningful results when applied for investigating other associations. This issue represents an important limitation of the proposed methodology and needs to be addressed in future research.

*Interpretational issues*. The point discussed previously is closely linked to the more general problem of interpretation of estimates of complex associations. Although the results illustrated in Section 5 are described on the original scale of the first-stage model, estimation is carried out in the multivariate dimension of the spline parameters. In practice, we read the association in the usual exposure–response frame, but we model it through coefficients of a function. We presuppose that these parameters, in this multi-study assessment, still preserve their interpretation and that the way we model the relationship between study-level variables and the multivariate distribution of the outcome parameters reflects the association of interest. If, for example, different combinations of parameters define exactly the same association, this link vanishes, and interpretation of the results would be less straightforward. This issue requires further consideration.

*Model selection and control for confounding*. The modelling framework presented in this contribution requires a complex two-stage model selection. In the first stage, the selection of a suitable model involves the choices for the function *s* to define the association and for the control for potential confounders. As previously discussed, this procedure is usually specific to the data, study design and regression method applied in the first stage. Also, this selection procedure should always be accompanied by a sensitivity analysis. The selection in the second stage is limited to the choice of the meta-predictors to be included in the meta-analytical model and may follow traditional methods used in regression analysis, exploiting the multivariate extension of tests and statistics defined in Section 4.3. However, further research is required to provide a general methodological approach for this two-stage selection procedure.

## 7. Discussion

In this contribution, we provide a methodological overview of the application of multivariate meta-analysis and meta-regression for the investigation of complex associations that are described by multiple parameters. The modelling framework we propose is general and potentially applicable to different research fields, such as multi-site clinical trials, multi-centre cohorts, studies on multiple cancer registries or multi-city time-series studies, each involving different first-stage regression models. The methodology requires the specification of a common function to model the association, which nevertheless may be selected among several potential alternatives, like splines, step functions, fractional polynomials or threshold functions, among others. The statistical framework is nested within that of the general linear mixed model, which offers the basis for the specification of tests and statistics for inferential procedures, model comparison and model checking.

Previous work explored methods to pool non-linear dose–response relationships within IPD two-stage analyses. A recent paper has proposed a method to obtain pooled estimates through a series of univariate meta-analyses of estimated effects on a grid of exposure values in order to re-construct the pooled non-linear relationships [52]. This flexible approach was previously developed and termed *meta-smoothing* in the context of multi-city time-series studies [53] and potentially allows the use of different study-specific functions to model the association. However, the meta-analytic estimates of the effects in the grid are treated as independent, although obtained from the same first-stage model, an approach that could affect the validity of the estimates for standard errors and confidence intervals. Although the framework we propose is limited by the definition of the same function in all the studies, it accommodates in an appropriate way the correlation between estimates from the first stage, establishing valid inferential procedures about the pooled association. Also, it formalizes the meta-analytic procedure within a proper statistical model, providing statistics and significance tests for model fit and comparison, heterogeneity and prediction. Other investigators have previously applied multivariate meta-analysis for IPD analysis of non-linear exposure–response relationships, although not focusing on methodological aspects. A recent paper has discussed the statistical methods for two-stage analysis of multi-site cohorts, also illustrating the use of multivariate meta-analysis for pooling dose–response associations that have been estimated using multiple categories [5]. Other examples include applications in multi-city time-series studies to assess potential non-linear effects of air pollution [7, 54, 55], using approaches similar to the example in Section 5.

Methods for obtaining pooled dose–response dependencies from published epidemiological studies, rather than IPD analyses, have been also investigated in previous research. Pioneering work [56, 57] described an analysis based on log-RR estimates for different exposure categories compared with a common reference, in which the whole within-study (co)variance matrix is reconstructed using ad hoc approximations. The estimates of linear and (optionally) quadratic terms were then combined using fixed meta-analytic methods and then the random counterpart based on method of moments. This approach has also been applied with splines or fractional polynomials to model non-linearity [58–60]. More recently, a general methodological treatment of the meta-analysis of published estimates for non-linear associations has been provided [61, 62], also on the basis of multivariate meta-analysis [63, 64].

The methodology illustrated here is not limited to model multi-parameterized non-linear exposure–response dependencies: investigators have also applied multivariate meta-analysis to synthesize survival curves [30, 65], longitudinal profiles [66], ROC curves [67] and heat wave effects [68]. Other studies have adopted multivariate meta-analysis to explore the distributed lag effects of air pollution [69–71] and temperature [72]. Finally, the same methods have also been applied to pool main and interaction terms across studies [57, 73].

In its traditional setting for pooling multiple health end points in randomized controlled trials, multivariate meta-analysis offers parameter estimates with better statistical properties, in particular a potentially increased precision from accommodating the estimated between-study covariance structure [11]. Nonetheless, the analysis could be carried out with multiple univariate meta-analysis, although often less efficiently. In the application we have described, instead, estimates of complex associations, such as those illustrated in Figures 2–6, cannot be provided by simple univariate models without important limitations or additional assumptions. In this context, multivariate meta-analysis offers clear advantages. As discussed in Section 4, this modelling framework can be seen as an example of a multivariate linear mixed model. The extensive body of research defining this statistical framework may therefore be exploited in this context, for example for the definition of tests discussed in Section 4.3. There are, of course, specific issues that deserve further research, for example statistics for heterogeneity, methods for handling missing correlations or a critical comparison of estimation methods. Other important issues specific to multivariate meta-analysis have been illustrated and discussed by Jackson and colleagues [11] and need to be addressed in future research in order to develop this methodology as a standard analytical tool.

## Software

All the analyses presented in this contribution are performed in R (version 2.15.0). The package mvmeta (version 0.2.4) is used to run multivariate meta-analysis and meta-regression. The package dlnm (version 1.6.0) [74] is used to specify the basis matrices for the quadratic spline for temperature and to predict and plot the results of a fitted model. The data are accessed through functions in the package NMMAPSlite. The R code to replicate all the results of Section 5 is available in the Supplementary Web Appendix, together with additional information on the package mvmeta. An alternative software implementation of multivariate meta-analysis is available in Stata[28, 40], whereas a SAS code has been previously presented [75].

## Footnotes

‡ Supporting information may be found in the online version of this article.
