# Supplementary figures and images for: Multivariate meta-analysis for non-linear and other multi-parameter associations
Source: Stat Med. 2012 Jul 16;31(29):3821–39. doi: 10.1002/sim.5471 (PMC3546395; doi:10.1002/sim.5471)

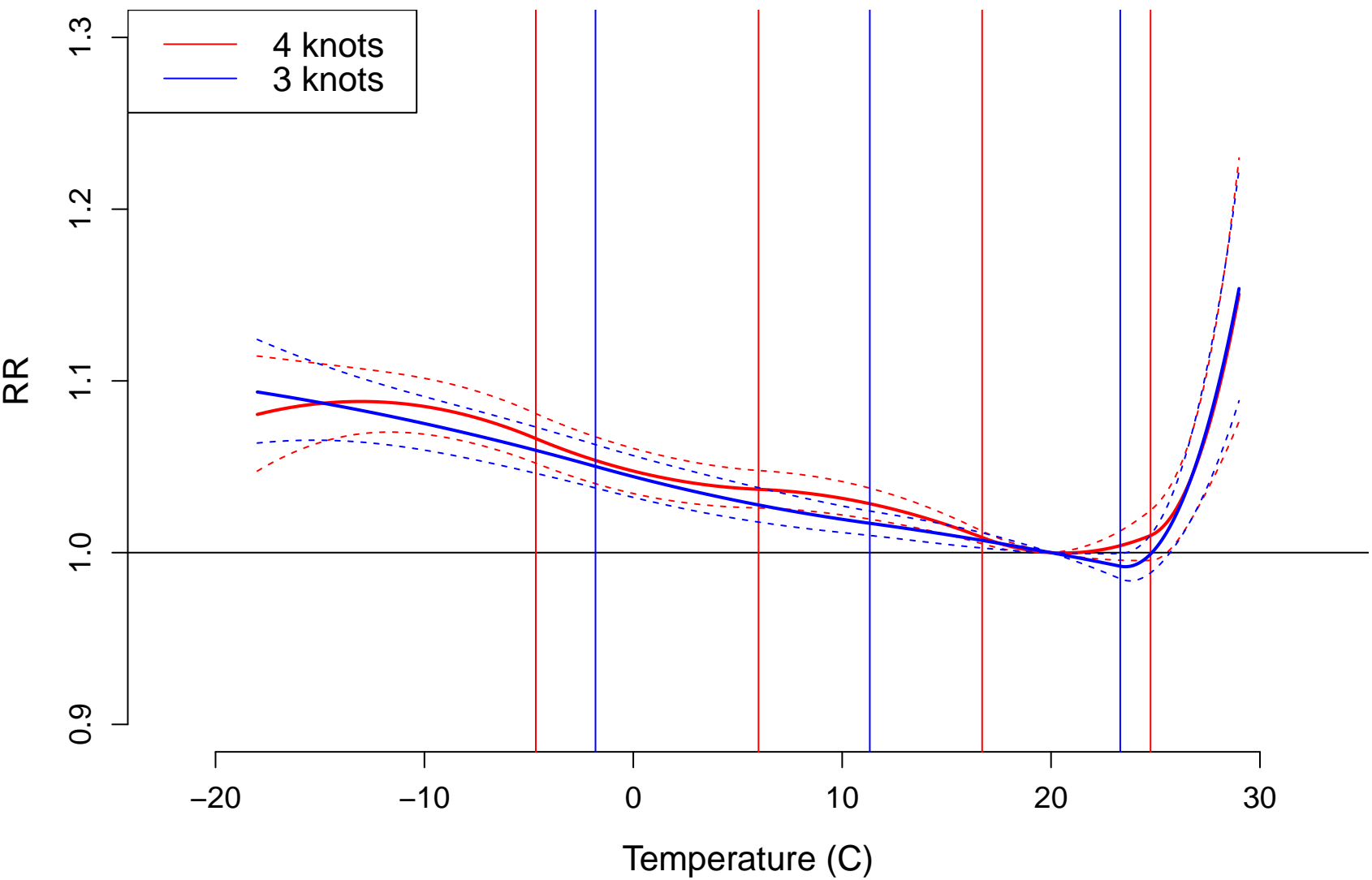

Supplement: Supplementary file 9 [file sim0031-3821-SD9.pdf]

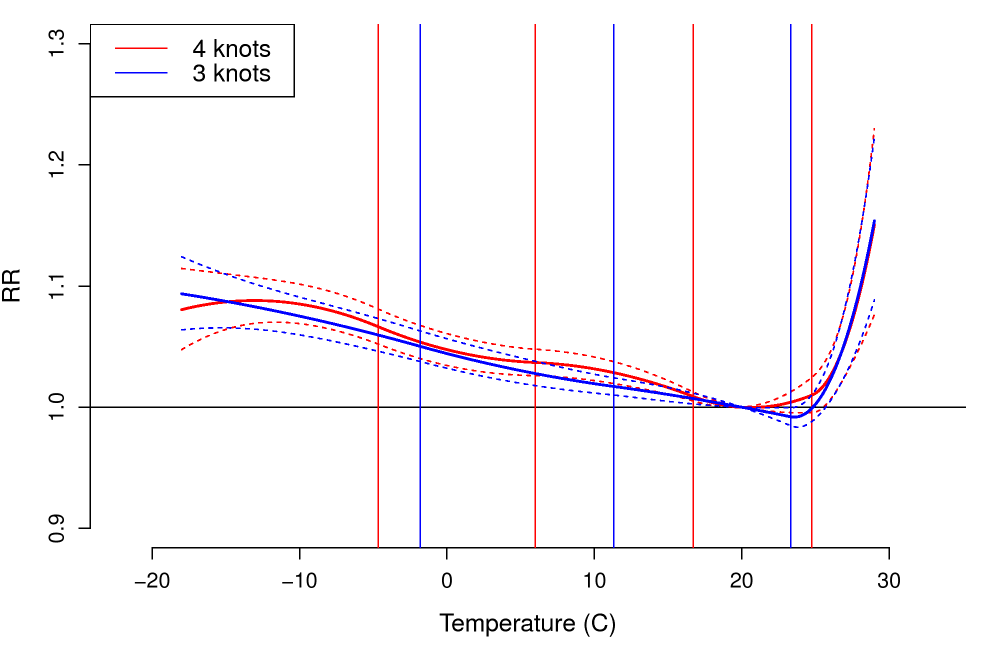

Supplement: Supplementary file 11 [file sim0031-3821-SD11.png]

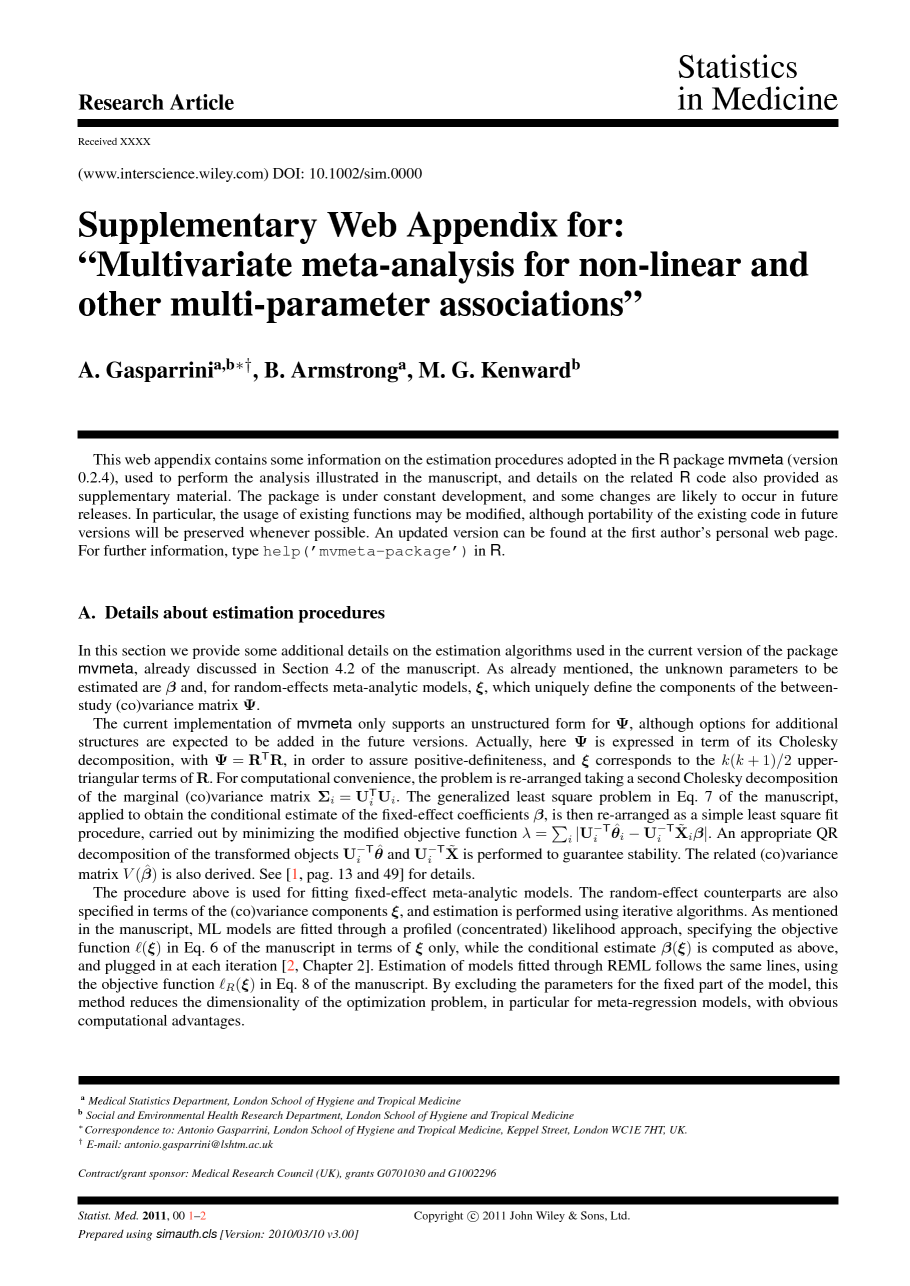

Supplement: Supplementary file 12 [file sim0031-3821-SD12.png]
