# Supplementary material for: Multivariate meta-analysis for non-linear and other multi-parameter associations
Source: Stat Med. 2012 Jul 16;31(29):3821–39. doi: 10.1002/sim.5471 (PMC3546395; doi:10.1002/sim.5471)
Supplement: Supplementary file 10 [file sim0031-3821-SD10.pdf]

Research Article

Statistics in Medicine

Received XXXX

(www.interscience.wiley.com) DOI: 10.1002/sim.0000

Supplementary Web Appendix for: "Multivariate meta-analysis for non-linear and other multi-parameter associations"

A. Gasparrini[a,b*†], B. Armstrong[a], M. G. Kenward[b]

This web appendix contains some information on the estimation procedures adopted in the R package mvmeta (version 0.2.4), used to perform the analysis illustrated in the manuscript, and details on the related R code also provided as supplementary material. The package is under constant development, and some changes are likely to occur in future releases. In particular, the usage of existing functions may be modified, although portability of the existing code in future versions will be preserved whenever possible. An updated version can be found at the first author's personal web page. For further information, type `help('mvmeta-package')` in R.

A. Details about estimation procedures

In this section we provide some additional details on the estimation algorithms used in the current version of the package mvmeta, already discussed in Section 4.2 of the manuscript. As already mentioned, the unknown parameters to be estimated are $\boldsymbol{\beta}$ and, for random-effects meta-analytic models, $\boldsymbol{\xi}$, which uniquely define the components of the between-study (co)variance matrix $\boldsymbol{\Psi}$.

The current implementation of mvmeta only supports an unstructured form for $\boldsymbol{\Psi}$, although options for additional structures are expected to be added in the future versions. Actually, here $\boldsymbol{\Psi}$ is expressed in term of its Cholesky decomposition, with $\boldsymbol{\Psi} = \mathbf{R}^\mathsf{T}\mathbf{R}$, in order to assure positive-definiteness, and $\boldsymbol{\xi}$ corresponds to the $k(k+1)/2$ upper-triangular terms of $\mathbf{R}$. For computational convenience, the problem is re-arranged taking a second Cholesky decomposition of the marginal (co)variance matrix $\boldsymbol{\Sigma}_i = \mathbf{U}_i^\mathsf{T}\mathbf{U}_i$. The generalized least square problem in Eq. 7 of the manuscript, applied to obtain the conditional estimate of the fixed-effect coefficients $\boldsymbol{\beta}$, is then re-arranged as a simple least square fit procedure, carried out by minimizing the modified objective function $\lambda = \sum_i |\mathbf{U}_i^{-\mathsf{T}}\hat{\boldsymbol{\theta}}_i - \mathbf{U}_i^{-\mathsf{T}}\tilde{\mathbf{X}}_i\boldsymbol{\beta}|$. An appropriate QR decomposition of the transformed objects $\mathbf{U}_i^{-\mathsf{T}}\hat{\boldsymbol{\theta}}$ and $\mathbf{U}_i^{-\mathsf{T}}\tilde{\mathbf{X}}$ is performed to guarantee stability. The related (co)variance matrix $V(\hat{\boldsymbol{\beta}})$ is also derived. See [1, pag. 13 and 49] for details.

The procedure above is used for fitting fixed-effect meta-analytic models. The random-effect counterparts are also specified in terms of the (co)variance components $\boldsymbol{\xi}$, and estimation is performed using iterative algorithms. As mentioned in the manuscript, ML models are fitted through a profiled (concentrated) likelihood approach, specifying the objective function $\ell(\boldsymbol{\xi})$ in Eq. 6 of the manuscript in terms of $\boldsymbol{\xi}$ only, while the conditional estimate $\boldsymbol{\beta}(\boldsymbol{\xi})$ is computed as above, and plugged in at each iteration [2, Chapter 2]. Estimation of models fitted through REML follows the same lines, using the objective function $\ell_R(\boldsymbol{\xi})$ in Eq. 8 of the manuscript. By excluding the parameters for the fixed part of the model, this method reduces the dimensionality of the optimization problem, in particular for meta-regression models, with obvious computational advantages.

[a] *Medical Statistics Department, London School of Hygiene and Tropical Medicine*
[b] *Social and Environmental Health Research Department, London School of Hygiene and Tropical Medicine*
[*] *Correspondence to: Antonio Gasparrini, London School of Hygiene and Tropical Medicine, Keppel Street, London WC1E 7HT, UK.*
[†] *E-mail: antonio.gasparrini@lshtm.ac.uk*

Contract/grant sponsor: Medical Research Council (UK), grants G0701030 and G1002296

Copyright © 2011 John Wiley & Sons, Ltd.

In the current implementation of mvmeta, the maximization of the objective functions is obtained through a quasi-Newton iterative algorithm, a variation of the Newton-Raphson method [3], exploiting the built-in R function `optim()`. Briefly, in the quasi-approach, the computation of the updated guess only requires the vector of first partial derivative of $\ell(\boldsymbol{\xi})$ or $\ell_R(\boldsymbol{\xi})$ with respect to $\boldsymbol{\xi}$, while using an approximation of the inverse of the Hessian, the matrix of second partial derivative, obtained from previous iterations. The equations for the vectors of first partial derivatives are provided in [4]. Convergence of Newton methods is heavily dependent on optimal starting values $\boldsymbol{\xi}^{[0]}$: these are provided by performing few runs of an iterative generalized least square algorithm [5, 6].

Missing values in the estimated outcome parameters or (co)variance matrix for study i are naturally handled in the optimization algorithms by excluding the corresponding entries of $\hat{\boldsymbol{\theta}}_i$ and rows of $\tilde{\mathbf{X}}_i$, although no missing parameter or element of the (co)variance matrices occurs in the analysis proposed in this paper.

B. The R code

The R scripts provided as supplementary material reproduce all the results illustrated in the manuscript, figures included. Although the code could have been written in a more concise and faster version, we have privileged clarity here. The R packages mvmeta, dlnm, tsModel, maps and NMMAPSlite, available in the R CRAN, need to be installed. The scripts are meant to be run consecutively. An updated version can be found at the first author's personal web page.

The first script is used to generate the data, producing a list of databases for the 108 NMMAPS cities and a database with the city-level meta-predictors used in multivariate meta-regression models. Additional meta-variables have been included, so the reader can extend the investigation. Note that the script takes several minutes to complete, as the data are downloaded by an external repository.

The second script selects the subset of 20 cities included in the analysis and defines some useful quantities.

The third script performs the model selection based on Q-AIC and identify the knots and *df* for the best model.

The fourth script performs the first-stage time-series Poisson model. It first produces the basis matrix for temperature using the function `onebasis()` in the package dlnm. Although this package is expressly meant to be used for distributed lag (non-linear) models, it is applied here to produce the basis for the temperature spline, which is automatically centered and conveniently lagged. In addition, as described below, other functions in the package dlnm help extracting the parameters from the fitted model, and facilitates the prediction and plotting of the estimated exposure-response relationships. After the Poisson models are fitted, the estimated parameters for temperature are extracted and stored, together with associated (co)variance matrices.

The fifth scripts runs the second-stage models, namely multivariate meta-analyses and meta-regressions, and computes the predicted effects. This step is carried out through the functions in mvmeta and dlnm. After the models have been fitted through the function `mvmeta()`, basis matrices are created to obtain the predictions for a set of values in a specific range of temperature, built by `onebasis()` using the same specifications as in the original basis used for estimation. The prediction of the average curve is computed through the function `crosspred()`, including the estimated outcome parameters from multivariate meta-analytic models. For meta-regression models, outcomes parameters and associated (co)variance matrices for specific percentiles of the meta-variables are predicted through the method function `predict()` for `mvmeta` objects, before the association is predicted on the original scale through `crosspred()`.

The sixth script produces the main results illustrated in the manuscript, including tables and the figures, conveniently saved as pdf files. The code exploits the `plot()` and `lines()` method functions for objects `crosspred` where the predictions have been saved, which facilitates the graphical representation.

The seventh script performs the analysis using a relative scale of temperature.

The eighth script provides additional results presented in the manuscript, in particular the comparison with REML models and results from multivariable multivariate meta-regression.

References

1. Wood SN. *Generalized Additive Models: an Introduction with R*. Chapman & Hall/CRC, 2006.
2. Pinheiro JC, Bates DM. *Mixed-Effects Models in S and S-PLUS*. Springer Verlag: New York, 2000.
3. Nocedal J, Wright SJ. *Numerical Optimization*. Springer: New York, 2006.
4. Lindstrom MJ, Bates DM. Newton-Raphson and EM algorithms for linear mixed-effects models for repeated-measures data. *Journal of the American Statistical Association* 1988; **83**(404):1014–1022.
5. Goldstein H. Multilevel mixed linear model analysis using iterative generalized least squares. *Biometrika* 1986; **73**(1):43.
6. Goldstein H. Restricted unbiased iterative generalized least-squares estimation. *Biometrika* 1989; **76**(3):622.

 Copyright © 2011 John Wiley & Sons, Ltd.
